# Blood biomarkers of Alzheimer's disease in the community: Variation by chronic diseases and inflammatory status

**DOI:** 10.1002/alz.13860

**Published:** 2024-05-08

**Authors:** Martina Valletta, Davide Liborio Vetrano, Debora Rizzuto, Bengt Winblad, Marco Canevelli, Sarah Andersson, Matilda Dale, Claudia Fredolini, Laura Fratiglioni, Giulia Grande

**Affiliations:** ^1^ Aging Research Center Department of Neurobiology, Care Sciences and Society Karolinska Institutet and Stockholm University Stockholm Sweden; ^2^ Stockholm Gerontology Research Center Stockholm Sweden; ^3^ Division of Neurogeriatrics, Department of Neurobiology, Care Sciences and Society Karolinska Institutet Solna Sweden; ^4^ Theme Inflammation and Aging, Karolinska University Hospital Huddinge Sweden; ^5^ Department of Human Neuroscience Sapienza University Rome Italy; ^6^ National Centre for Disease Prevention and Health Promotion Italian National Institute of Health Rome Italy; ^7^ Affinity Proteomics Stockholm, Science for Life Laboratory, Department of Protein Science, School of Engineering Sciences in Chemistry, Biotechnology and Health (CBH) Royal Institute of Technology (KTH) Solna Sweden

**Keywords:** Alzheimer's disease, blood biomarkers, chronic diseases, dementia, inflammation, population‐based study

## Abstract

**INTRODUCTION:**

We explored the variations of blood biomarkers of Alzheimer's disease (AD) by chronic diseases and systemic inflammation.

**METHODS:**

We explored the association of AD blood biomarkers with chronic diseases and systemic inflammation (interleukin‐6 [IL‐6]), in 2366 dementia‐free participants of the Swedish National Study on Aging and Care‐in Kungsholmen, using quantile regression models.

**RESULTS:**

A greater number of co‐occurring chronic diseases was associated with higher concentrations of phosphorylated‐tau 181 (p‐tau181), total‐tau (t‐tau), neurofilament light chain (NfL), and glial fibrillary acidic protein (GFAP) (*p* < 0.01). Anemia, kidney, cerebrovascular, and heart diseases were associated with variations in the levels of AD blood biomarkers. Participants in the highest (vs. lowest) interleukin‐6 (IL‐6) tertile had higher NfL concentration. Systemic inflammation amplified the associations between several chronic diseases and p‐tau181, t‐tau, NfL, and GFAP.

**DISCUSSION:**

In the community, the concentration of AD blood biomarkers varies in relation to medical conditions and systemic inflammation. Recognizing these influences is crucial for the accurate interpretation and clinical implementation of blood biomarkers.

**Highlights:**

- Participants with a complex clinical profile (i.e., multiple co‐occurring diseases or specific disease combinations) display elevated levels of AD blood‐biomarkers.
- Anemia, heart, cerebrovascular, and kidney diseases are associated with variations is the levels of AD blood biomarkers in cognitively intact older adults.
- Systemic inflammation amplifies the association between several chronic diseases and AD blood biomarkers.

## 1 BACKGROUND

Biomarkers of Alzheimer's disease (AD) measured in blood were shown to correlate with cerebrospinal fluid (CSF)^1^ and cerebral positron emission tomography (PET)^1^, ^2^, ^3^ biomarkers of AD pathology, and to accurately predict all‐cause and AD dementia.^3^, ^4^, ^5^, ^6^ These promising results, coupled with the minimal invasiveness and lower cost of blood biomarker measurement, suggest the potential for their clinical implementation in early AD detection. However, most prior studies on blood biomarkers of AD were conducted in specialized clinical settings, and their findings may not be directly translated to the general population.^7^ Indeed, in the community individuals are more heterogeneous and often present with multiple chronic diseases that could potentially influence the blood concentration of these biomarkers, posing challenges to their clinical interpretation.^7^, ^8^ It is therefore important to evaluate factors that may influence the levels of AD blood biomarkers in population‐based studies, that are more representative of real‐world settings.

Several diseases were previously associated with blood concentrations of AD biomarkers in population‐based cohorts. Among them, chronic kidney disease was consistently shown to be associated with a higher level of circulating AD biomarkers, whereas obesity was associated with lower biomarker concentrations.^9^, ^10^, ^11^, ^12^, ^13^, ^14^, ^15^ More sparsely, cardiovascular diseases were also associated with elevated levels of AD biomarkers.^9^, ^10^, ^13^ The reasons underlying such associations are not fully understood.

Whether other conditions, as well as the co‐occurrence of multiple diseases, may influence the blood levels of these biomarkers in a real‐world setting is still under‐investigated. Similarly, the potential role played in these associations by systemic low‐grade inflammation requires proper exploration. In fact, systemic inflammation might be interpreted as a proxy of an accelerated aging process and is often associated with higher clinical complexity^16^ in older adults.

Using data from a population‐based cohort of 2366 dementia‐free individuals, aged 60 years or older, from the Swedish National Study on Aging and Care in Kungsholmen (SNAC‐K), this study aimed to explore (1) the association of demographics, apolipoprotein E (*APOE)* genotype and chronic diseases, alone and in combination, with the concentrations of several blood biomarkers of AD; and (2) whether the associations between chronic diseases and AD blood biomarkers varied by systemic inflammatory status.

## 2 METHODS

### 2.1 Study population

We used data from the SNAC‐K,^17^ an ongoing longitudinal population‐based study. At baseline (2001–2004), 3363 individuals aged 60 years or older from the Kungsholmen district of Stockholm were randomly enrolled (73.3% participation rate). Participants were recruited based on their age across 11 different age cohorts (i.e., age 60, 66, 72, 78, 81, 84, 87, 90, 93, 96, 99+) and were followed‐up at intervals of 6 (<78 years old) or 3 (≥78 years old) years. From baseline SNAC‐K participants, we excluded those with dementia (*n* = 240). Further, we excluded 10 participants missing information on dementia, 665 without available blood samples, and 82 missing at least one of the measured AD biomarkers, leaving a final analytical sample of 2366 participants.

All phases of SNAC‐K were approved by the ethical committee at Karolinska Institutet and the Regional Ethical Review Board in Stockholm, Sweden (ethical permit for baseline: KI 01‐114). All participants provided written informed consent to participate in the study.

The results of this study are reported in keeping with the STROBE recommendations.^18^

### 2.2 Data collection

At baseline, participants underwent medical examination, nurse interview, and cognitive assessment conducted by trained staff. Data on demographics were obtained during nurse interviews. Educational level was categorized into elementary, high school, or university and above. Mini‐Mental State Examination (MMSE) was used as a measure of global cognition.^19^

### 2.3 Blood biomarkers and APOE genotyping

Peripheral venous blood samples were collected at baseline (fasting was not compulsory) and upon centrifugation serum aliquots were stored at the Karolinska Institutet Bio Bank at −80°C in cryogenic storage vials until analysis. For AD blood biomarkers, protein quantification was conducted at the Affinity Proteomics Stockholm Unit (SciLifeLab). Serum neurofilament light chain (NfL) and glial fibrillary acidic protein (GFAP) were measured using Simoa Neuro 2‐plex B Kit. Serum amyloid‐β40 (Aβ40), amyloid‐β42 (Aβ42), and total‐tau (t‐tau) were measured using Simoa Neuro 3‐plex A Kit​. Serum phosphorylated‐tau181 (p‐tau181) was measured using Simoa pTau‐181 Advantage V2 Kit. For each kit, 25 µL of sample were diluted 1:4 and the assays were performed according to manufacturer instructions. The Quanterix instrument provides AEB (average enzyme per bead) values for calibrators, controls, and samples. Curve‐fitting, extrapolation of concentrations and graphical representation, are automatically performed within the Quanterix SR‐X software using the calibrators and a four‐parameter logistic (4PL) curve fit. Data below the limit of detection were replaced, using a not missing at random strategy, through single‐value imputation, with a value of 0 (imputed measurements: *n* = 6 for Aβ42, 15 for p‐tau181 and 15 for t‐tau).

Serum concentration of interleukin‐6 (IL‐6) was used as a measure of systemic inflammation. Serum IL‐6 was measured at Accelerator Laboratory Services, Quanterix Corp., in Billerica (MA, USA), using Simoa CorPlex Human Cytokine Panel 1 on the Quanterix SP‐XTM imaging and analysis platform.

DNA was extracted from peripheral blood samples for *APOE* genotyping. Participants were classified as *APOE‐ ε4* carriers if they had at least one *APOE‐ ε4* allele, or non‐carriers if they had none.

### 2.4 Chronic diseases and cardiovascular risk factors

Chronic diseases and cardiovascular risk factors were identified at baseline by the examining physician through a previously reported comprehensive assessment.^20^ Diagnoses were based on self‐reports, medical examination, laboratory and instrumental tests, medication use, medical charts and data from the Swedish National Patient Register (inpatient and outpatient care). Diseases were coded following the International Classification of Diseases, 10^th^ revision (ICD‐10). In the present study we investigated: hypertension, dyslipidemia, body mass index [BMI] ≥30, diabetes, chronic kidney disease, heart diseases (i.e., ischemic heart disease, atrial fibrillation, heart failure), cerebrovascular disease, cancer, and anemia. This selection was based on previous findings and knowledge gaps, as well as considering the high prevalence of such conditions in the older population.

**RESEARCH IN CONTEXT**

1. **Systematic review**: The authors reviewed the literature (PubMed and Embase) and found few population‐based studies reporting an association between some chronic diseases and altered levels of Alzheimer's disease (AD) blood biomarkers. However, data on the co‐occurrence of multiple diseases and on the involvement of systemic low‐grade inflammation in these associations are still lacking.
2. **Interpretation**: In the community, the concentration of AD blood biomarkers varies in relation to multiple factors. Among chronic diseases, the strongest associations were found for anemia, heart, cerebrovascular, and kidney diseases, alone and—even more—when combined. Systemic inflammation was associated with elevated levels of neurofilament light chain (NfL) and amplified the associations between several chronic diseases and elevated AD blood biomarkers.
3. **Future directions**: Future studies are needed to clarify the mechanisms underlying the associations between chronic diseases and AD blood biomarkers and ascertain whether these influences can affect the diagnostic and prognostic performance of blood biomarkers in detecting impending dementia.

### 2.5 Statistical analyses

We used Kruskal‐Wallis test to compare biomarkers’ concentrations between groups, and Spearman correlations to explore between‐biomarker correlations. Blood biomarkers were transformed into z‐scores based on baseline mean and standard deviation of the study population, facilitating comparison between coefficients.

Quantile regression models on the 50^th^ (median) percentile were used to examine the associations of demographics, *APOE* genotype, chronic diseases, and IL‐6 (predictors) with AD blood biomarkers (outcomes). The associations were tested using separate models (i.e., one for each predictor) adjusted for age, sex, and education. Analyses for the association between IL‐6 and blood biomarkers of AD were further adjusted for the number of chronic diseases.

Additionally, we tested the interaction between IL‐6 concentration (continuous values, centered around the median) and chronic diseases in relation to AD blood biomarkers and repeated the analyses for the association between chronic diseases and AD blood biomarkers stratifying by IL‐6 concentration (third vs. first and second tertiles).

In a sensitivity analysis, we explored the associations between demographics, APOE genotype, chronic diseases, IL‐6 (predictors) and AD blood biomarkers (outcomes), adjusted for age, sex, and education, excluding participants with MMSE score below 27 (*n *= 250) or missing data on MMSE (*n* = 4),^21^ to further restrict the analysis to a cognitively intact population.

A two‐tailed *p*‐value < 0.05 was considered statistically significant in all analyses. The statistical analyses were performed using Stata version 18 (StataCorp, Texas, USA); GraphPad Prism 9 was used for graphical representations.

## 3 RESULTS

### 3.1 Characteristics of the study population

**FIGURE 1:**
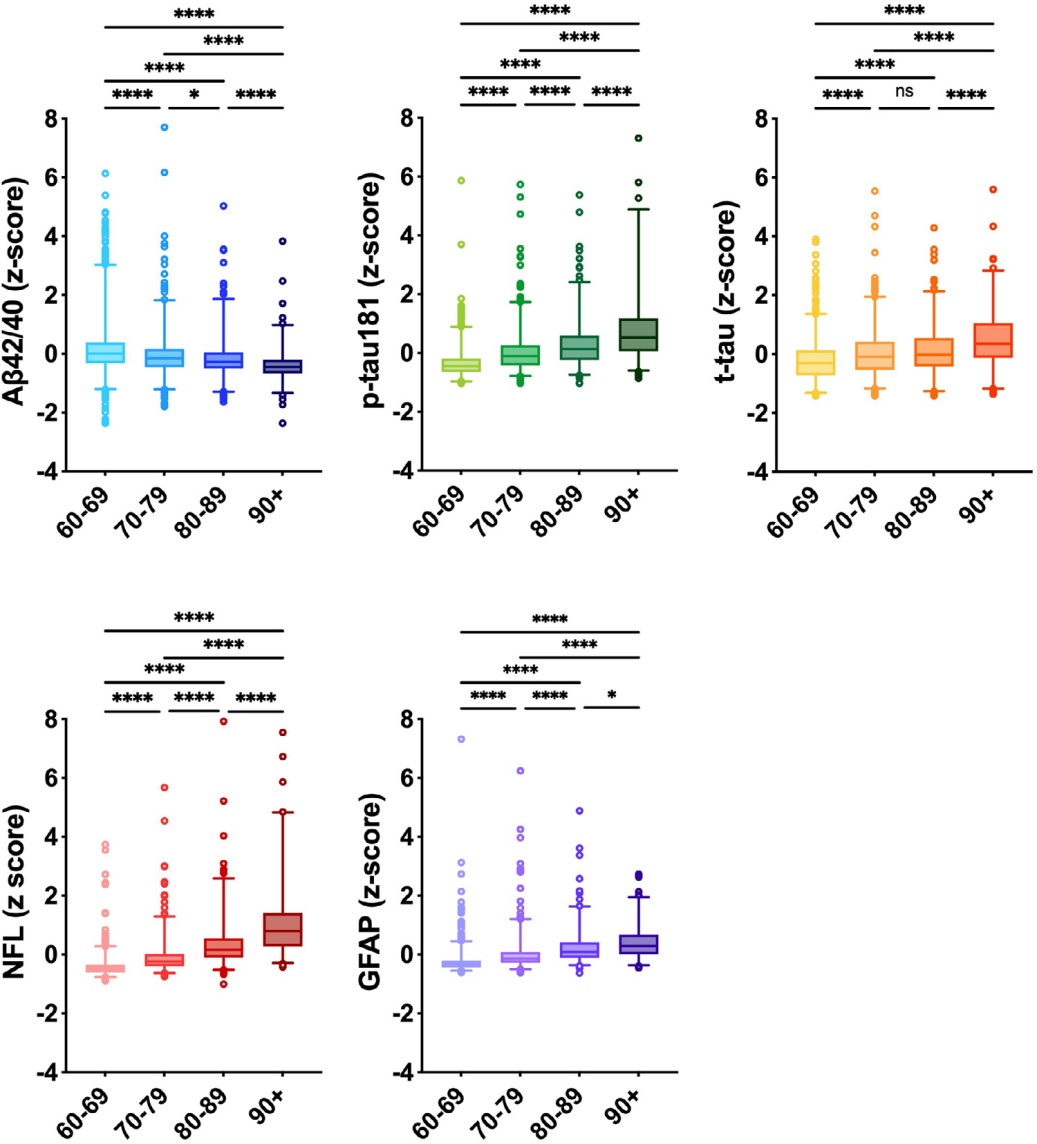
Distribution of blood biomarkers of Alzheimer's disease (z‐scores) by age groups. Box plots show the median (central line) and interquartile range (box) as well as the 2.5^th^ and 97.5^th^ percentiles (whiskers). *p* values are derived from Kruskal‐Wallis test; ns: non‐significant, *: < 0.05; **: < 0.01; ***: < 0.001; ****: < 0.0001. Aβ42/40, amyloid‐beta 42/40; GFAP, glial fibrillary acidic protein; NfL, neurofilament light chain; p‐tau181, phosphorylated‐tau181; t‐tau, total‐tau. Some outliers were not represented for graphical purposes (*n* = 2 for Aβ42/40 ratio, 3 for p‐tau181, 2 for t‐tau, 6 for NfL, and 5 for GFAP).

Characteristics of the study population are reported in Table 1. Overall, median age was 72.2 years, 61.7% were females, 35.8% had an educational level of university or above, and 29.4% were *APOE‐ε4* carriers. Older participants had higher concentrations of all the biomarkers, except for the Aβ42/40 ratio, which was lower in the older age groups (Figure 1).

**TABLE 1:** Baseline characteristics of the study population.

Parameter	Overall (*n* = 2366)
Demographics	
Age	72.2 (60.9–81.1)
Sex (female), *n* (%)	1461 (61.7)
Education (university), *n* (%)	848 (35.8)
*APOE* (at least one ε4 allele), *n* (%)	675 (29.4)
Cardiovascular risk factors	
Hypertension, *n* (%)	1624 (68.6)
Dyslipidemia, *n* (%)	1189 (50.3)
BMI ≥30, *n* (%)	306 (12.9)
Diabetes, *n* (%)	212 (9.0)
Chronic diseases	
No. of chronic diseases	3.0 (2.0–5.0)
Chronic kidney disease, *n* (%)	775 (32.8)
Ischemic heart disease, *n* (%)	323 (13.7)
Atrial fibrillation, *n* (%)	198 (8.4)
Heart failure, *n* (%)	190 (8.0)
Cerebrovascular disease, *n* (%)	139 (5.9)
Cancer	198 (8.4)
Anemia, *n* (%)	243 (10.3)
Blood biomarkers	
Aβ42/40	0.06 (0.05–0.07)
p‐tau181 (pg/mL)	1.2 (0.7–1.8)
t‐tau (pg/mL)	0.8 (0.5–1.2)
NfL (pg/mL)	18.1 (12.6–28.6)
GFAP (pg/mL)	122.3 (81.4–190.4)
IL‐6 (pg/mL)	1.6 (0.9–2.8)

*Note*: Data are reported as: median (Q1‐Q3) or *n* (%). Missing: 1 in education, 71 in APOE, 10 in IL‐6.

Abbreviations: *APOE*, apolipoprotein E; Aβ42/40, amyloid‐beta 42/40; BMI, body mass index; GFAP, glial fibrillary acidic protein; IL‐6, interleukin‐6.; NfL, neurofilament light chain; p‐tau181, phosphorylated‐tau 181; t‐tau, total tau.

Table S1 reports the comparison between dementia‐free SNAC‐K participants with and without AD biomarkers. Overall, participants without AD blood biomarkers were older, more likely to be female and had a lower educational level than those with available biomarkers; they also presented a higher number of chronic diseases and a lower MMSE score.

### 3.2 Correlations between biomarkers

**FIGURE 2:**
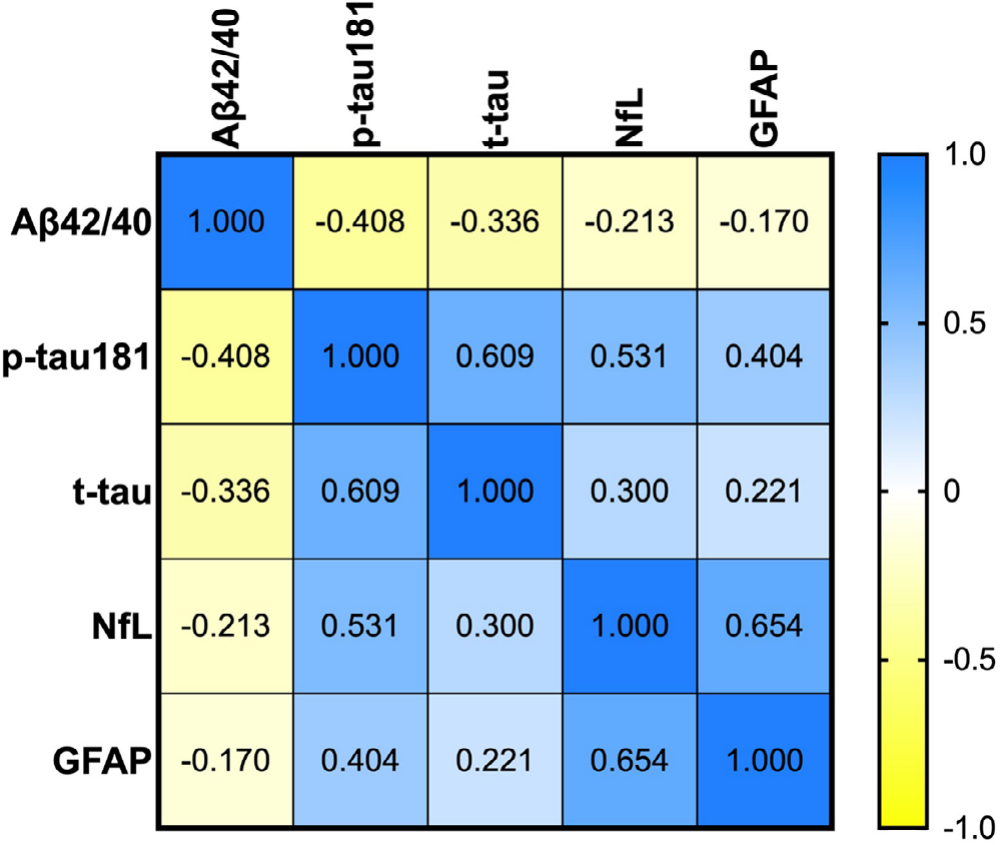
Correlation matrix showing Spearman's correlations between blood biomarkers of Alzheimer's disease. Aβ42/40, amyloid‐beta 42/40; GFAP, glial fibrillary acidic protein; NfL, neurofilament light chain; p‐tau181, phosphorylated‐tau181; t‐tau, total‐tau.

All AD biomarkers were correlated to each other (*p* value < 0.001 for all, Figure 2); the strongest correlations were observed between NfL and GFAP (Spearman's rho = 0.654), p‐tau181 and t‐tau (Spearman's rho = 0.609), and p‐tau181 and NfL (Spearman's rho = 0.531). Both sex and age affected only slightly between‐biomarker correlations (Figure S1).

### 3.3 Demographics and *APOE* genotype

In multi‐adjusted models, older age was associated with lower Aβ42/40 ratio and higher concentrations of p‐tau181, t‐tau, NfL, and GFAP (Figure S2). Females presented with higher Aβ42/40 ratio (β 0.09, 95% CI 0.04, 0.14), lower levels of p‐tau181 (β −0.12, 95% CI −0.17, −0.07), and higher levels of GFAP (β 0.08, 95% CI 0.05, 0.10) than males. The Aβ42/40 ratio was lower (β −0.15, 95% CI −0.20, −0.10), while p‐tau181 concentration was higher (β 0.08, 95% CI 0.03, 0.14) in *APOE‐ε4* carriers compared with non‐carriers (Figure S2).

### 3.4 Chronic diseases and cardiovascular risk factors

**FIGURE 3:**
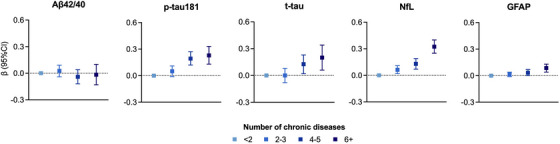
Associations between number of chronic diseases and blood biomarkers of Alzheimer's disease (AD). β coefficients with 95% confidence intervals are derived from quantile regression models on the 50th (median) percentile, adjusted for age, sex and education. Blood biomarkers of AD were z‐scored. *p* for trend: Aβ42/40: *p* 0.405; p‐tau181: *p* < 0.001; t‐tau: *p* 0.007; NfL: *p* < 0.001, GFAP *p* 0.001. Aβ42/40, amyloid‐beta 42/40; GFAP, glial fibrillary acidic protein; NfL, neurofilament light chain; p‐tau181, phosphorylated‐tau181; t‐tau, total‐tau.

**FIGURE 4:**
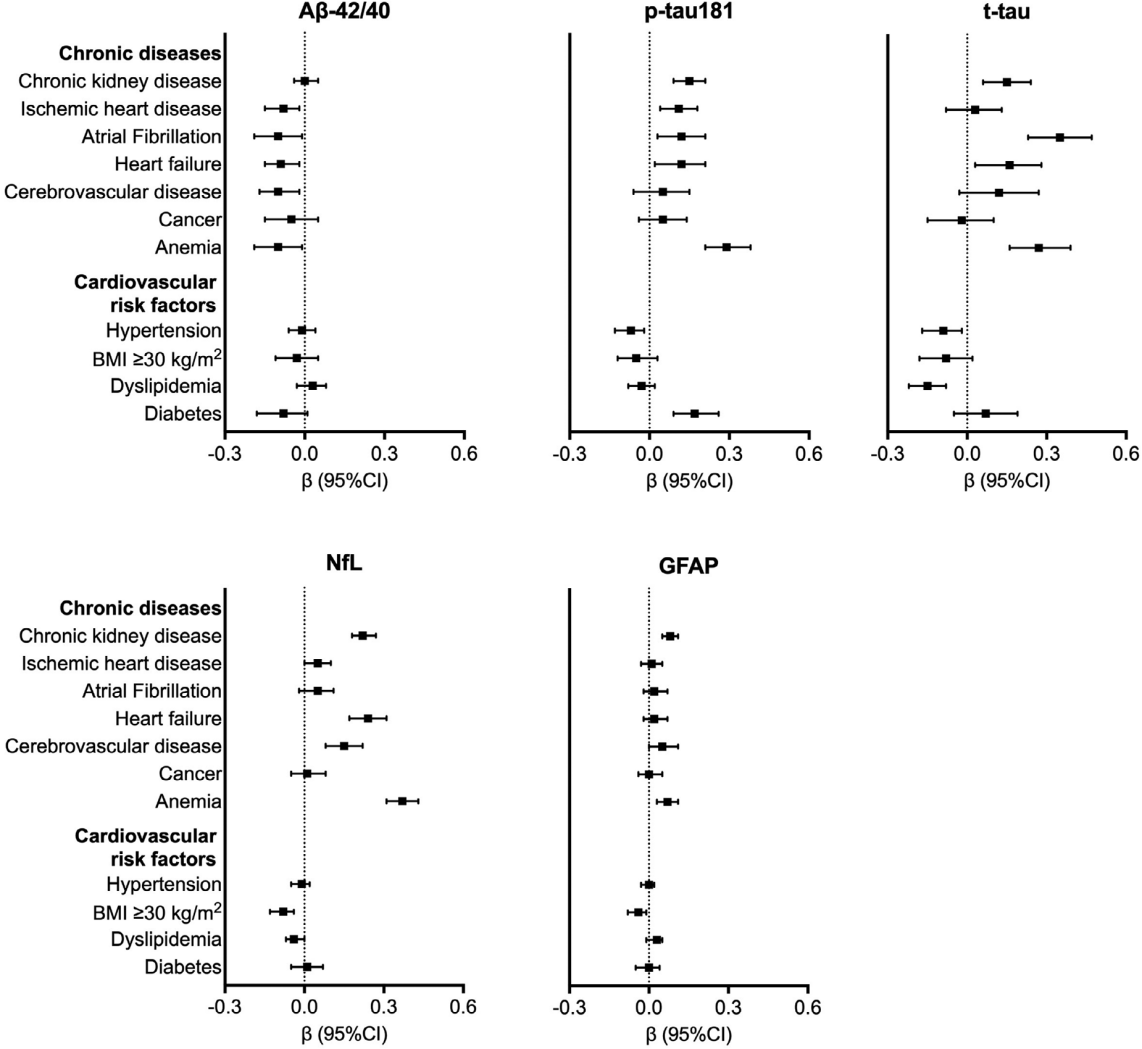
Associations of chronic diseases and cardiovascular risk factors with blood biomarkers of Alzheimer's disease (AD). β coefficients with 95% confidence intervals are derived from quantile regression models on the 50th (median) percentile, adjusted for age, sex, and education. Blood biomarkers of AD were z‐scored. Aβ42/40, amyloid‐beta 42/40; GFAP, glial fibrillary acidic protein; NfL, neurofilament light chain; p‐tau181, phosphorylated‐tau181; t‐tau, total‐tau.

We found an association between a higher number of co‐occurring chronic diseases and higher concentrations of p‐tau181, t‐tau, NfL, and GFAP, following a dose‐response relationship (*p* for trend < 0.01) (Figure 3). Considering individual chronic diseases, participants with cerebrovascular disease showed lower Aβ42/40 ratio and higher concentrations of NfL and GFAP, and those with heart diseases showed lower Aβ42/40 ratio and higher levels of p‐tau181, t‐tau, and NfL, compared with participants free from these diseases (Figure 4). Additionally, chronic kidney disease was associated with elevated levels of p‐tau181, t‐tau, NfL, and GFAP. Variations in the levels of all the examined biomarkers were observed in presence of anemia (Figure 4).

Looking at specific disease combinations, the concentrations of p‐tau181, t‐tau, NfL, and GFAP were even further elevated when two among chronic kidney disease, anemia, and heart diseases co‐occurred in the same person (Figure S3).

We found an inverse association between cardiovascular risk factors and several blood biomarkers of AD. Participants with hypertension had lower concentrations of p‐tau181 and t‐tau compared with those with normal/low blood pressure, and those with BMI ≥30 had lower levels of NfL and GFAP than those with BMI < 30. We also found an association between dyslipidemia and lower concentrations of t‐tau and NfL (Figure 4). Diabetes was instead associated with elevated levels of p‐tau181 (Figure 4).

### 3.5 Systemic inflammation

A higher concentration of IL‐6 (highest vs. lowest tertile) was associated with higher levels of NfL (β 0.03, 95% CI 0.00, 0.06). Several significant interactions were found between IL‐6 concentration (centered around the median) and chronic diseases in relation to AD blood biomarkers. Specifically, we observed significant interactions between IL‐6 and chronic kidney disease in relation to NfL (β 0.02, 95% CI 0.02–0.03, *p* < 0.001), between IL‐6 and cerebrovascular disease in relation to p‐tau181 (β 0.04, 95% CI 0.02–0.06, *p* 0.001) and GFAP (β 0.02, 95% CI 0.01–0.03, *p* 0.001) and between IL‐6 and anemia in relation to p‐tau181 (β 0.02, 95% CI 0.01–0.03, *p* 0.003), NfL (β 0.02, 95% CI 0.01–0.03, *p* < 0.001) and GFAP (β 0.01, 95% CI 0.00–0.02, *p* 0.001).

**FIGURE 5:**
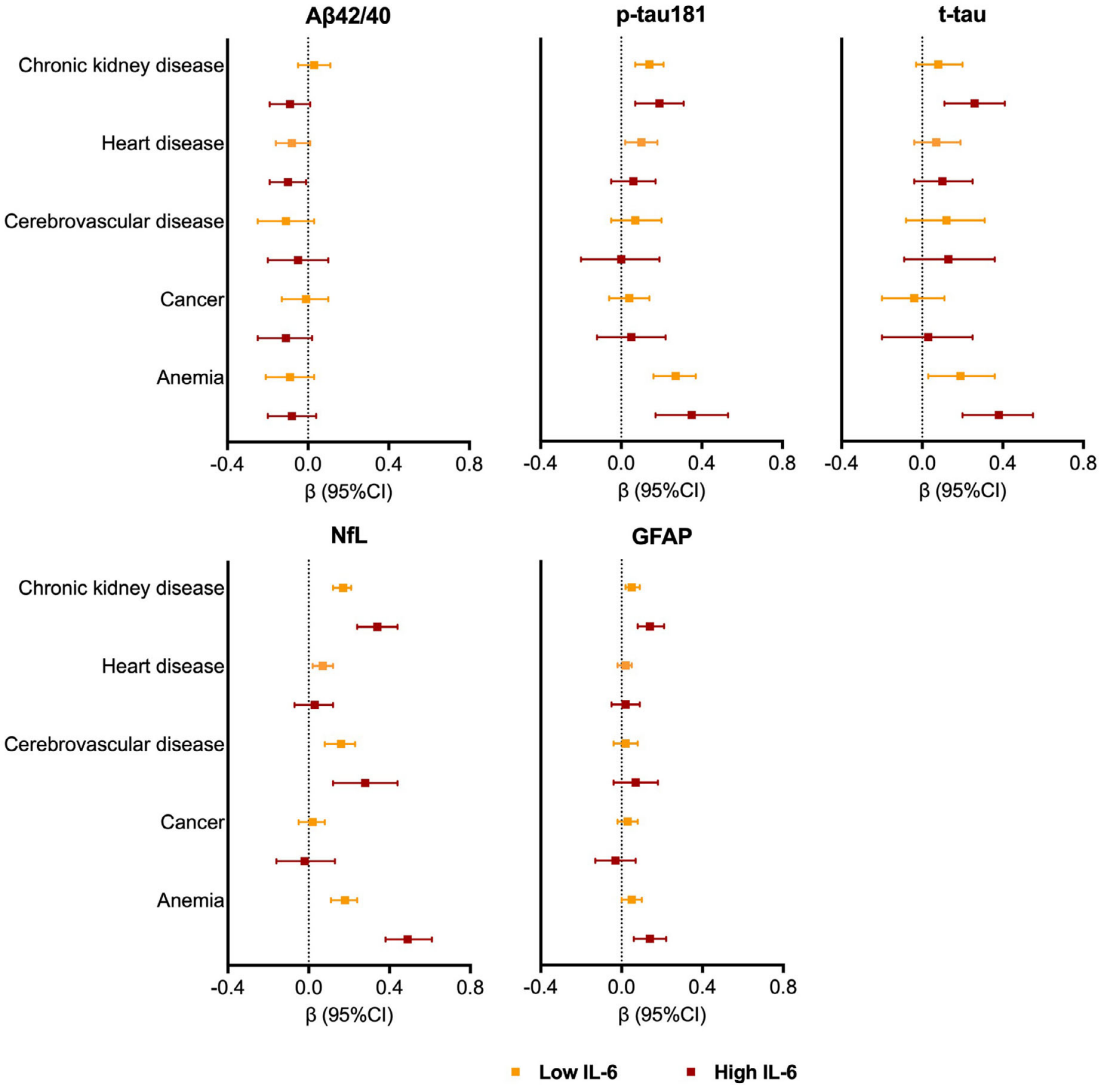
Associations between chronic diseases and blood biomarkers of Alzheimer's disease (AD), stratified by IL‐6 levels. β coefficients with 95% confidence intervals are derived from quantile regression models on the 50th (median) percentile adjusted for age, sex, and education. Blood biomarkers of AD were z‐scored. Low and high levels of IL‐6 were categorized based on tertiles (low IL‐6 = first and second IL‐6 tertiles, IL‐6 ≤ 2.2 pg/mL; high IL‐6 = third IL‐6 tertile, IL‐6 > 2.2 pg/mL). Heart disease includes atrial fibrillation, heart failure and ischemic heart disease. Aβ42/40, amyloid‐beta 42/40; GFAP, glial fibrillary acidic protein; IL‐6, interleukin 6; NfL, neurofilament light chain; p‐tau181, phosphorylated‐tau181; t‐tau, total‐tau.

In analyses stratified by IL‐6 levels (Figure 5), the association between chronic kidney disease and concentration of NfL, t‐tau, and GFAP was stronger in participants with high levels of IL‐6 than in those with low IL‐6. Participants in the high IL‐6 subgroup also exhibited a stronger association between cerebrovascular disease and levels of NfL and GFAP than those in the low IL‐6 subgroup. Finally, the association between anemia and the concentrations of all the biomarkers, except for the Aβ42/40 ratio, was stronger in participants with high IL‐6 than in those with low IL‐6 (Figure 5).

### 3.6 Sensitivity analysis

Most of the associations between demographics, *APOE* genotype, chronic diseases, IL‐6 levels, and blood biomarkers of AD did not change after the exclusion of participants with a MMSE score below 27 (Figure S4 and S5). However, the associations between atrial fibrillation and Aβ42/40 ratio (β −0.09, 95% CI −0.19, 0.02), stroke and Aβ42/40 ratio (β −0.08, 95% CI −0.20, 0.04), heart failure and p‐tau181 (β 0.08, 95% CI −0.02, 0.19), heart failure and t‐tau (β 0.11, 95% CI −0.03, 0.26) were non‐significant in this subgroup. On the other hand, a significant association emerged between diabetes and the Aβ42/40 ratio (β −0.10, 95% CI −0.20, −0.01) and between female sex and t‐tau (β 0.08, 95% CI 0.01, 0.16).

## 4 DISCUSSION

In this large population‐based cohort of dementia‐free older adults, the concentration of blood biomarkers of AD varied in relation to multiple factors, including demographics, *APOE* genotype, medical conditions, and systemic inflammation. Identifying those factors that may influence the levels of blood biomarkers of AD is a critical step in the roadmap toward their implementation in clinical practice.^8^, ^22^

While previous studies on AD blood biomarkers were primarily conducted in specialized clinical settings, limited evidence arises from community‐dwelling cohorts, which are more representative of the real‐world setting. We not only confirm but also extend previous findings by assessing diseases that were not considered before, such as anemia, investigating the co‐occurrence of multiple diseases and exploring the influence of systemic inflammation on AD blood biomarkers concentration. In this exploratory study, we were able to consider a large set of variables that — directly or indirectly — could alter the blood concentration of five different AD blood biomarkers.

Based on our findings, older participants exhibited lower Aβ42/40 ratio and higher concentrations of the other biomarkers than their younger counterparts and *APOE‐ε4* carriers had a lower Aβ42/40 ratio and higher p‐tau181 concentration than non‐carriers. These results align with previous studies^4^, ^9^, ^10^, ^14^ and may reflect the increased neuropathological burden associated with aging and *APOE‐ε4* carriership, which in turn influence the risk of dementia.

Beyond age and genetic predisposition, we explored several medical conditions in relation to the circulating level of blood biomarkers. We observed a dose‐response relationship between the number of co‐occurring chronic diseases and the levels of all the examined biomarkers, except for the Aβ42/40 ratio. Similarly, two studies from the Mayo Clinic Study of Aging found elevated levels of blood biomarkers of AD in participants with higher Charlson comorbidity index.^9^, ^10^ These results align with the concept that a higher disease burden may contribute to brain pathology and accelerate cognitive decline.^23^ Notably, previous studies found that a high chronic disease burden was associated with neuroimaging markers of neurodegeneration (i.e., brain atrophy and reduced metabolism)^24^, ^25^, ^26^ but not with amyloid deposition.^25^, ^27^ The association between chronic diseases and AD blood biomarkers may arise from various mechanisms. Certain diseases may exert an impact on brain structure and function, increasing neuropathology and dementia risk, while others may interfere peripherally with the distribution and metabolism of blood biomarkers.

Delving further into individual diseases, we found that cerebrovascular, heart and kidney diseases, as well as anemia, were those most frequently associated with variations in biomarker levels. The heart‐brain connection is known to play a role in dementia development.^28^ Heart diseases are linked to cerebral hypoperfusion,^29^ embolization, cerebral small vessels disease,^30^ as well as brain^31^, ^32^ and hippocampal^33^ atrophy. The variation in the levels of AD blood biomarkers associated with heart diseases might reflect these mechanisms.

Elevated levels of blood biomarkers of AD in relation to renal function have been repeatedly observed^9^, ^10^, ^13^, ^34^, ^35^ and may be attributed to reduced renal clearance. In line with this hypothesis, Stocker et al. found that impaired kidney function was associated with higher concentrations of blood biomarkers of AD but not with higher dementia risk.^15^ This suggests that kidney impairment may alter the level of AD blood biomarkers through a peripheral mechanism rather than through an effect on neuropathology. Nevertheless, a link between impaired kidney function and higher dementia risk has also been sparsely reported.^36^ Future studies should delve deeper into the link between kidney function, AD blood biomarkers and dementia risk and assess whether different biomarkers’ cutoffs should be applied for individuals with impaired kidney function.

Ours is the first study that reports an association between anemia and variations in the levels of blood biomarkers of AD. Anemia is a highly prevalent condition in the older population, affecting 10% of individuals over age 65^37^ and up to 30% of those over age 80.^38^ There is some evidence that individuals with anemia are at higher risk of dementia^39^, ^40^ and that low hemoglobin is associated with cerebral hypoxia and neuroimaging pathological changes.^39^ At the same time, in the older population, anemia may be the result of multiple other conditions, including chronic diseases (e.g., CKD, cancer) and chronic inflammation, thus proxying a greater clinical complexity encountered in some older adults.^37^ The impact of anemia on blood biomarkers of AD and on dementia risk needs to be further investigated.

Older adults are known to suffer from multiple chronic conditions,^20^ which tend to cluster in the same individual following shared underlying biological mechanisms and/or risk factors.^41^ We explored how the levels of AD blood biomarkers varied in relation to combinations of anemia, heart, and chronic kidney diseases and observed that biomarkers’ concentrations were even higher when two of these diseases, instead of one alone, co‐occurred in the same individual. This finding points to the need to further investigate the impact of co‐occurring chronic diseases on AD pathology.

In our study, cardiovascular risk factors, apart from diabetes, were associated with lower concentrations of several biomarkers. Previous studies reported an inverse association between BMI and p‐tau181, p‐tau217, and NfL,^9^, ^10^, ^14^, ^42^ which is hypothesized to be due to higher blood volume and fat mass, leading to a different distribution of the biomarkers in obese individuals.^7^, ^42^ On the other hand, our findings on the relation between hypertension and AD biomarkers are less clear, as in another population‐based study high blood pressure was associated with higher levels of t‐tau and amyloid markers.^10^ The relationship between blood pressure and brain aging is intricate; in fact, hypertension ‐mid‐life hypertension in particular‐ is well‐known to contribute to AD pathology, but there is evidence that late‐life hypotension is also a risk factor for AD pathology,^43^, ^44^ and some studies suggest that antihypertensive medications may reduce AD risk.^43^ Beyond the effects of blood pressure on neuropathology and dementia risk, the association between hypertension and biomarker levels may also reflect peripheral mechanisms (e.g., higher blood volume in individuals with hypertension). Future studies are needed to disentangle the complex link between blood pressure, blood biomarkers of AD, and dementia risk.

For the first time, our study highlighted the role of low‐grade systemic inflammation in the association between chronic diseases and AD blood biomarkers concentration. Systemic inflammation is a driver of the aging process,^45^ as well as both trigger and manifestation of several diseases,^16^, ^46^ and can be interpreted as a marker of higher clinical complexity in older adults. Also, the imbalance between pro‐inflammatory and anti‐inflammatory agents that occurs with aging (i.e., inflammaging) can ultimately contribute to neurodegeneration and dementia development.^47^, ^48^ Our study revealed an association between systemic inflammation and elevated levels of NfL. In addition, we found an interplay between certain chronic diseases and inflammation, ultimately influencing blood biomarkers’ concentration. Specifically, systemic inflammation amplified the association of chronic kidney disease, cerebrovascular disease and anemia with elevated blood biomarkers of AD. Conversely, systemic inflammation did not modify the relationship between heart diseases and blood biomarkers of AD, suggesting that other factors may drive this association.

Taken as a whole, these findings highlight that many features need to be considered in the interpretation of these biomarkers; special attention must be given to the clinical complexity that some individuals might display.

### 4.1 Limitations and strengths

Some aspects concerning the study must be considered. First, its cross‐sectional design restricts our possibility to speculate on the temporal and causal relation between the examined factors and the levels of the biomarkers. Moreover, AD biomarkers were measured in serum, while plasma biomarkers are more widely used. The concentrations of the biomarkers tend to be lower in serum than in plasma, however, serum biomarkers of AD have been already used in other studies to predict dementia development^49^, ^50^ and have shown a high correlation with plasma biomarkers and a similar diagnostic accuracy.^1^, ^51^ Further, SNAC‐K includes older adults who live in a central district in Stockholm and who are generally wealthy, fit, and healthy. This might limit the generalizability of our findings to other populations. However, similar results were reported in other population‐based studies conducted in different countries.^10^, ^15^ Additionally, among SNAC‐K participants, those with available AD blood biomarkers at baseline were younger and had fewer chronic diseases compared to those for whom blood biomarkers were not available. It is plausible to believe that this selection bias towards a relatively healthier sample may have resulted in an underestimation of the observed associations. Our study has some important strengths. We were able to measure five different blood biomarkers of AD and one marker of inflammation in a large and well‐characterized population‐based sample of more than 2000 dementia‐free participants. Furthermore, SNAC‐K participants undergo a comprehensive assessment that provides information on many variables of interest including demographics, *APOE* genotype, risk factors, and chronic diseases.

### 4.2 Conclusions

In the community, the concentration of blood biomarkers of AD varies in relation to multiple factors. Understanding these influences is crucial for their optimal interpretation. Future studies should delve deeper into the mechanisms underlying these associations and ascertain whether these factors can affect the diagnostic and prognostic performance of AD blood biomarkers.

## AUTHOR CONTRIBUTIONS

Martina Valletta, Davide Liborio Vetrano, and Giulia Grande contributed to the conception and design of the study. Claudia Fredolini, Matilda Dale, and Sarah Andersson conducted the biomarkers’ analyses. Martina Valletta conducted the statistical analyses. Martina Valletta conducted the literature search. All authors contributed to interpretation of the results. Martina Valletta drafted the first version of the manuscript. All authors critically revised the manuscript for important intellectual content. All authors made a significant contribution to the research and the development of the manuscript and approved the final version for publication.

## CONFLICT OF INTEREST STATEMENT

The authors declare no conflict of interest. Author disclosures are available in the Supporting information.

## CONSENT STATEMENT

All participants provided written informed consent to participate in the study.

## Supporting information

Supporting Information

Supporting Information
