# Supplementary material for: Blood biomarkers of Alzheimer's disease in the community: Variation by chronic diseases and inflammatory status
Source: Alzheimers Dement. 2024 May 8;20(6):4115–25. doi: 10.1002/alz.13860 (PMC11180869; doi:10.1002/alz.13860)
Supplement: Supplementary file 2 — Supporting Information [file ALZ-20-4115-s001.docx]

**Table S1.** Baseline characteristics of SNAC-K dementia-free participants according to availability of blood biomarkers of Alzheimer’s disease at baseline

|  | **Overall**  **(N= 3113)** | **Biomarkers non-available**  (**N=747**) | **Biomarkers available**  (**N=2366**) | **p-value** |
| --- | --- | --- | --- | --- |
| **Age** | 73.6 (10.7) | 77.0 (10.4) | 72.5 (10.5) | <0.001 |
| **Sex (Female)** | 1,972 (63.3%) | 511 (68.4%) | 1,461 (61.7%) | <0.001 |
| **Education** |  |  |  | <0.001 |
| - **Elementary** | 492 (15.8%) | 136 (18.2%) | 356 (15.0%) |  |
| - **High school** | 1,544 (49.6%) | 383 (51.3%) | 1,161 (49.1%) |  |
| - **University** | 1,063 (34.1%) | 215 (28.8%) | 848 (35.8%) |  |
| ***APOE* (at least one ε4 allele)** | 817 (28.9%) | 142 (26.5%) | 675 (29.4%) | 0.19 |
| **Number of chronic diseases** | 3.9 (2.4) | 4.4 (2.6) | 3.8 (2.4) | <0.001 |
| **MMSE score** | 28.5 (2.3) | 28.1 (3.0) | 28.6 (2.0) | <0.001 |

Data are presented as mean (SD) for continuous measures, and n (%) for categorical measures. Missing data: 14 in education, 282 in *APOE*, 9 in MMSE score. Abbreviations: *APOE*: Apolipoprotein E; MMSE: mini mental state examination.

**Figure S1.** Correlation matrix showing Spearman’s correlations between blood biomarkers of Alzheimer’s disease, stratified by sex and age groups.

Abbreviations: Aβ42/40: amyloid-beta 42/40; p-tau181: phosphorylated-tau181; t-tau: total-tau; NfL: neurofilament light chain; GFAP: glial fibrillary acidic protein.

**Figure S2.** Associations of demographic characteristics and *APOE* genotype with the concentrations of blood biomarkers of Alzheimer’s disease.

**
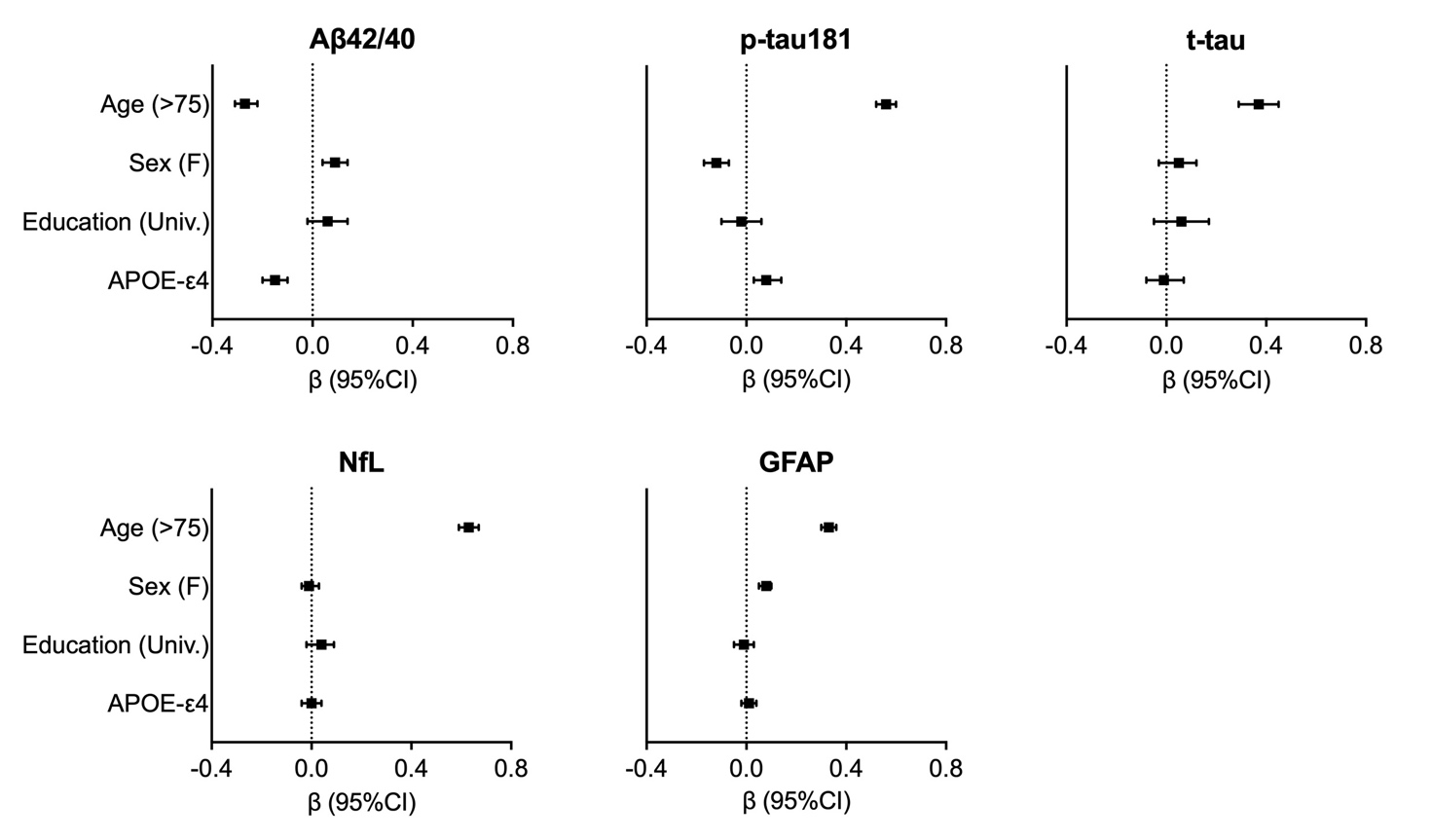
**

β coefficients with 95% confidence intervals are derived from quantile regression models on the 50^th^ (median) percentile adjusted for age, sex and education. Blood biomarkers were z-scored. Abbreviations: Aβ42/40: amyloid-beta 42/40; p-tau181: phosphorylated-tau181; t-tau: total-tau; NfL: neurofilament light chain; GFAP: glial fibrillary acidic protein; *APOE*: Apolipoprotein E.

**Figure S3.** Associations of combinations of anemia, chronic kidney disease and heart disease (i.e., at least one between atrial fibrillation, ischemic heart disease and heart failure) with the concentrations of blood biomarkers of Alzheimer’s disease.


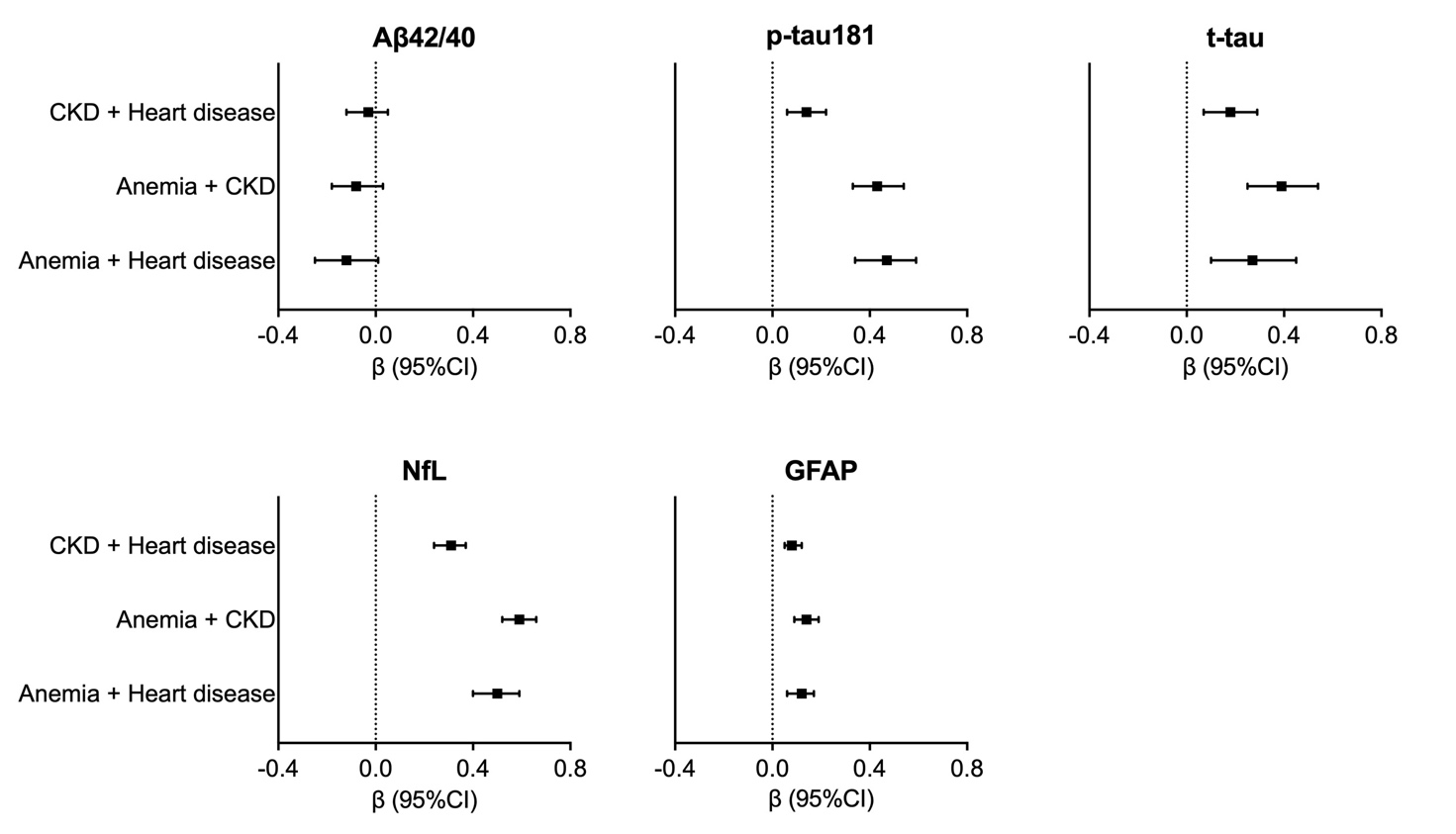


β coefficients with 95% confidence intervals are derived from quantile regression models on the 50^th^ (median) percentile adjusted for age, sex and education. Blood biomarkers were z-scored. Abbreviations: Aβ42/40: amyloid-beta 42/40; p-tau181: phosphorylated-tau181; t-tau: total-tau; NfL: neurofilament light chain; GFAP: glial fibrillary acidic protein; CKD: chronic kidney disease.

**Figure S4** Associations between demographics, *APOE* genotype and blood biomarkers of Alzheimer’s disease in participants with MMSE score ≥ 27.


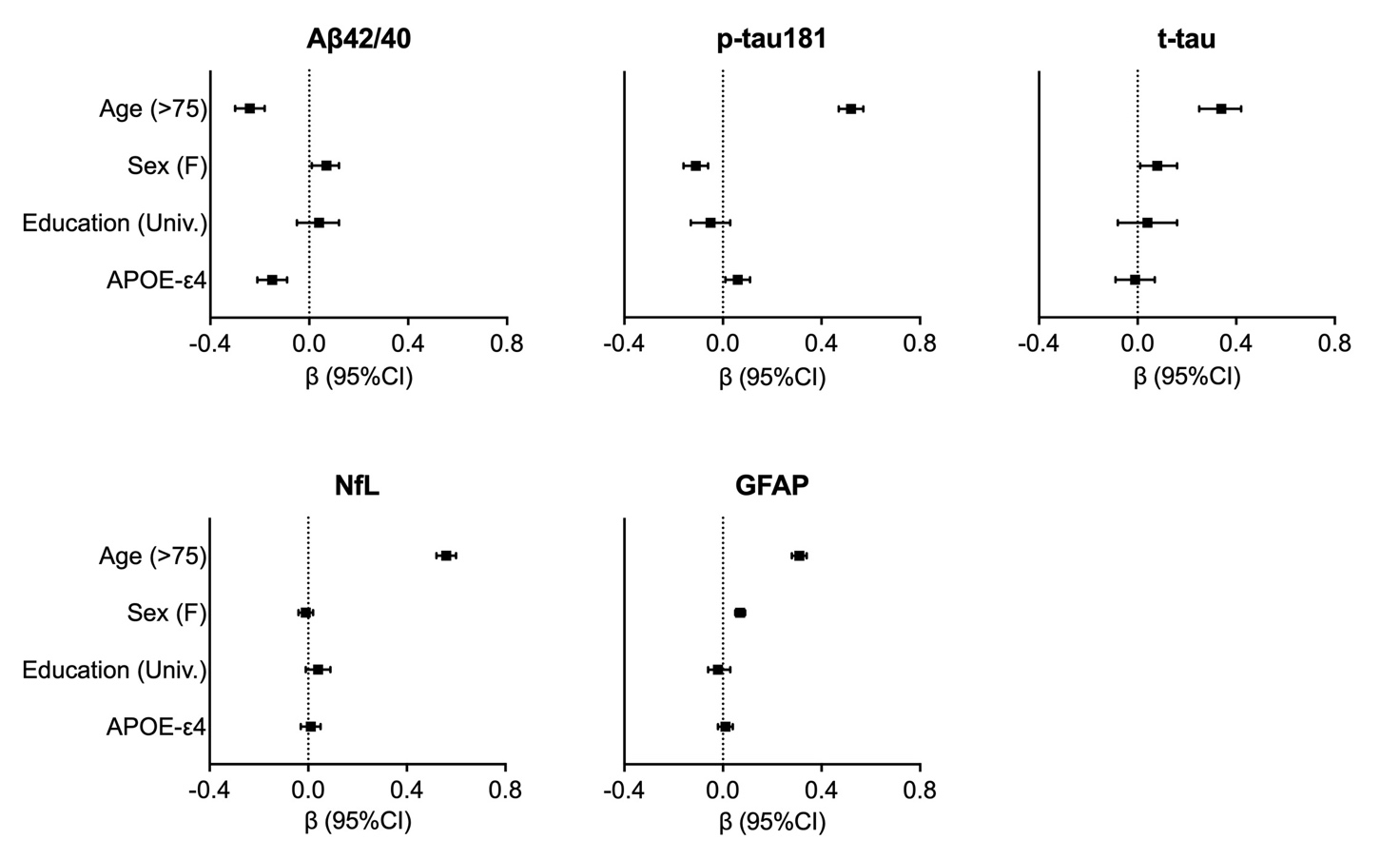


β coefficients with 95% confidence intervals are derived from quantile regression models on the 50^th^ (median) percentile adjusted for age, sex and education. Blood biomarkers of AD were z-scored. Abbreviations: *APOE*: apolipoprotein E; Aβ42/40: amyloid-beta 42/40; p-tau181: phosphorylated-tau181; t-tau: total-tau; NfL: neurofilament light chain; GFAP: glial fibrillary acidic protein.

**Figure S5** Associations between chronic diseases, cardiovascular risk factors, concentration of IL-6 (lowest tertile as reference) and blood biomarkers of Alzheimer’s disease in participants with MMSE score ≥ 27.


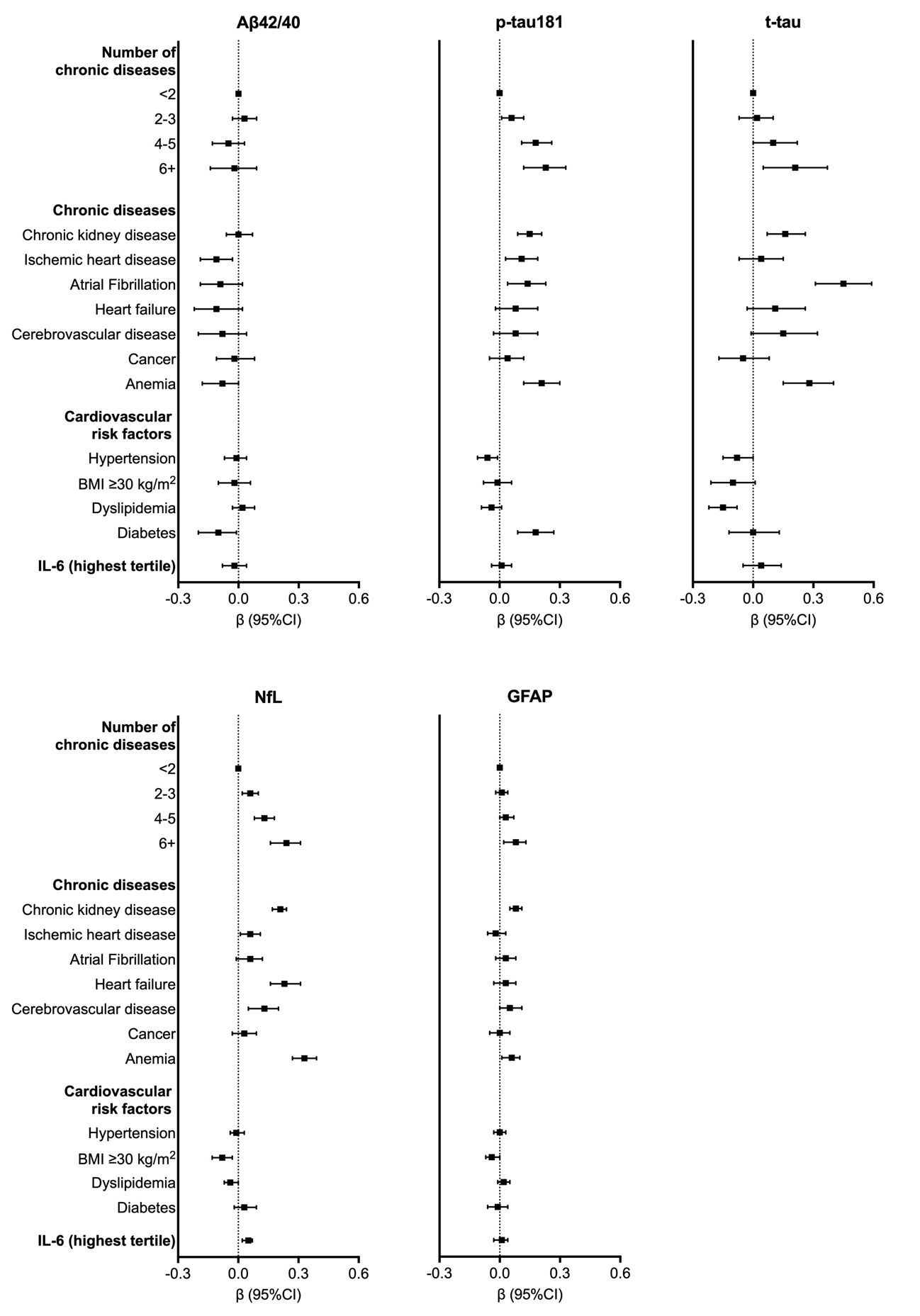


β coefficients with 95% confidence intervals are derived from quantile regression models on the 50^th^ (median) percentile adjusted for age, sex and education. Blood biomarkers of AD were z-scored. Abbreviations: Aβ42/40: amyloid-beta 42/40; p-tau181: phosphorylated-tau181; t-tau: total-tau; NfL: neurofilament light chain; GFAP: glial fibrillary acidic protein; IL-6: interleukin-6; BMI: body mass index.
